# Single-crystalline aluminum film for ultraviolet plasmonic nanolasers

**DOI:** 10.1038/srep19887

**Published:** 2016-01-27

**Authors:** Bo-Tsun Chou, Yu-Hsun Chou, Yen-Mo Wu, Yi-Cheng Chung, Wei-Jen Hsueh, Shih-Wei Lin, Tien-Chang Lu, Tzy-Rong Lin, Sheng-Di Lin

**Affiliations:** 1 Department of Electronics Engineering, National Chiao Tung University, Hsinchu, Taiwan; 2 Institute of Lighting and Energy Photonics, National Chiao Tung University, Tainan, Taiwan; 3 Department of Photonics, National Chiao Tung University, Hsinchu, Taiwan; 4 Department of Mechanical and Mechatronic Engineering, National Taiwan Ocean University, Keelung, Taiwan; 5 Department of Electrical Engineering, National Central University, Chungli, Taiwan; 6 Institute of Optoelectronic Sciences, National Taiwan Ocean University, Keelung, Taiwan

## Abstract

Significant advances have been made in the development of plasmonic devices in the past decade. Plasmonic nanolasers, which display interesting properties, have come to play an important role in biomedicine, chemical sensors, information technology, and optical integrated circuits. However, nanoscale plasmonic devices, particularly those operating in the ultraviolet regime, are extremely sensitive to the metal and interface quality. Thus, these factors have a significant bearing on the development of ultraviolet plasmonic devices. Here, by addressing these material-related issues, we demonstrate a low-threshold, high-characteristic-temperature metal-oxide-semiconductor ZnO nanolaser that operates at room temperature. The template for the ZnO nanowires consists of a flat single-crystalline Al film grown by molecular beam epitaxy and an ultrasmooth Al_2_O_3_ spacer layer synthesized by atomic layer deposition. By effectively reducing the surface plasmon scattering and metal intrinsic absorption losses, the high-quality metal film and the sharp interfaces formed between the layers boost the device performance. This work should pave the way for the use of ultraviolet plasmonic nanolasers and related devices in a wider range of applications.

Plasmonic devices have attracted much attention in the past decade, owing to their nanoscale size and high-speed operation. In recent years, various plasmonic devices have been proposed and demonstrated, including plasmonic gas detectors^1^, chemical sensors^2^,^3^, photovoltaic devices^4^,^5^,^6^,^7^, biomedical sensors^8^,^9^,^10^,^11^,^12^, superlenses^13^,^14^,^15^,^16^,^17^, optical trapping devices^18^,^19^, optical tweezers^20^,^21^,^22^,^23^, information technology^24^,^25^, optical integrated circuits^25^,^26^,^27^,^28^ and nanoscale coherent emitters^29^,^30^,^31^,^32^,^33^,^34^,^35^. In these plasmonic devices, the metal layer plays a critical role in determining the device performance. Gold, silver, and aluminum are the metals used frequently to generate plasmons in the visible and infrared spectral regime^4^,^9^,^12^,^15^,^29^,^32^,^36^,^37^,^38^,^39^. In terms of device fabrication, gold is the most ideal metal, as it is stable in air. However, its interband transition at approximately 2.3 eV makes it lossy and unsuitable to be used in applications corresponding to wavelengths shorter than 540 nm^37^,^40^. Silver has been employed in plasmonic devices to be used in the visible-to-ultraviolet regime. Owing to its strong interband absorption for wavelengths smaller than ~350 nm, silver exhibits significant intrinsic ohmic damping losses in the ultraviolet regime. In comparison to gold and silver, metallic aluminum is a cost-effective and abundant material that is used widely in modern semiconductor fabrication processes, and it also exhibits plasmonic properties superior to those of noble metals in the ultraviolet regime. Furthermore, aluminum films are stable in air and compatible with current CMOS technology; this is advantageous for integrating plasmonic devices with silicon-based electronic and photonic circuits^26^,^41^,^42^. Therefore, it is widely accepted that aluminum is the most promising metal for ultraviolet plasmonics devices^31^,^43^. On the other hand, it has been demonstrated that ultra-smooth single-crystalline metal films are crucial for fabricating high-definition plasmonic nanostructures^37^,^44^, and they also help improve the performances of optical antennae and plasmonic nanolasers^29^,^32^,^37^.

Nevertheless, it is challenging to grow large-area, flat, and single-crystalline aluminum films, denoted as SC-Al hereafter, as their growth process is highly sensitive to the surface conditions and morphology. Molecular beam epitaxy (MBE) is one of the most promising methods for growing such aluminum films, because it involves an ultrahigh vacuum, which provides a very clean environment and because the native oxide on the substrate is removed easily in the chamber just before aluminum film growth. Previously, several growth methods based on MBE have been used to grow aluminum films on As-stabilized AlAs surfaces^45^, thermal-anneal-induced^46^ and grown on silicon (111) substrates^47^. However, the single-crystalline aluminum films, which had thicknesses of as much as 200 nm, were quite rough. Rough surfaces are unsuitable for nanoscale plasmonic devices, as they increase the surface plasmon scattering rate and reduce the propagation length. Herein, by using a minimum-migration method^48^,^49^, we report the successful growth of highly flat single-crystalline aluminum films with a root-mean-square roughness of 0.44 nm on GaAs substrates by using MBE. The large-area growth of the films is attributable to the gallium-rich and flat nature of the substrates. Further, the film growth rate is high, and the process is highly reproducible. Thus, this process should aid the development of ultraviolet plasmonic devices.

Further, we use ultraviolet plasmonic nanolasers to highlight the effects of the quality of the metal film on plasmonic device performance. This is because nanolasers could provide a unique setting for the manipulation of light via the confinement of the electromagnetic field to a size well below the diffraction limit. In addition, they are an ideal nanoplatform for investigating how light interacts with nanomaterials^50^,^51^,^52^. Furthermore, reducing plasmonic losses is a key issue related to plasmonic nanolasers^31^,^32^,^37^,^38^, particularly in the ultraviolet regime. The lasing wavelength is on the nanoscale; hence, the plasmonic losses are highly sensitive to the degree of metal crystallization^32^, the lateral correlation length of the grain boundaries^39^ and the surface morphology^31^. Here, we first show that the MBE-grown aluminum film boosts nanolaser performance by minimizing the metal-related intrinsic damping and scattering losses. The plasmonic lasing mechanism can be explained on the basis of the amplification of the surface plasmons (SPs) by the stimulated emission radiation principle^53^, in a manner analogous to that for conventional photon lasers. The excitons in the gain medium are excited and nonradiatively transfer their energy to resonant surface plasmons SPs providing amplification channels. Since the modal volume (*V*_*m*_) is very small, a plasmonic cavity with a small *V*_*m*_ value (~ λ^3^/10 to λ^3^/1000)^29^,^30^,^31^,^32^,^33^,^35^,^50^,^54^ is beneficial for ensuring a high Purcell factor (*F*_p_) and efficiently boosting the energy transfer process. Therefore the population inversion of excitons can be realized readily, as this is highly advantageous for lowering the lasing threshold.

In this work, we use the metal-oxide-semiconductor (MOS) structure^29^,^31^,^32^,^33^ to fabricate ultraviolet nanolasers on a single-crystalline aluminum film. The key points for realizing these ultraviolet MOS nanolasers are as follows. To begin with, the use of a high-quality, single-crystalline metal film is crucial, as it can efficiently reduce the metallic losses and provide high conductivity for enhancing the degree of optical confinement and prolonging the propagating length of the SPs. Next, a flat surface drastically lowers the scattering loss of the SPs. Finally, close contact at the planar metal/oxide and oxide/semiconductor interfaces greatly lowers the scattering loss, and, more importantly, promotes the exciton-SP energy transfer process, thus ensuring that the gain is high enough to compensate for the losses and for achieving lasing^29^,^31^. As is described below, we could successfully fabricate ultralow-threshold, high-temperature-stable, and room-temperature ultraviolet nanolasers. These plasmonic nanolasers consist of zinc oxide (ZnO) nanowires placed on an aluminum film; an Al_2_O_3_ spacer layer is used to form the MOS structure. The extremely flat single-crystalline aluminum film is grown on a carefully treated GaAs surface by MBE. For comparison, we also fabricate a polycrystalline Al film, denoted as PC-Al, by e-gun evaporation (See Supporting Information for details).Furthermore, an ultrasmooth Al_2_O_3_ spacer layer is deposited on the SC-Al and PC-Al films by atomic layer deposition (ALD) to form the crucial interface for ensuring a flat interface and close contact between the metal and the semiconductor. The ZnO nanowires serve as the gain medium in the ultraviolet regime, since the large exciton binding energy and oscillator strength of ZnO aid the coupling between the excitons and SPs and allow for room-temperature operation^55^. Further, the hexagonal cross-sections of the ZnO nanowires allow for close contact. The experimental results underline the importance of using ultrasmooth, high-quality, and single-crystalline Al films as well as that of ensuring flat and sharp interfaces between the various layers in realizing low-threshold, room-temperature ultraviolet nanolasers.

Figure 1 (a,b) show 5 × 5 μm^2^ top-view atomic force microscopy (AFM) images of the SC-Al and PC-Al films in air, respectively. The root-mean-square roughness of the SC-Al film is 0.44 nm, which is 5.2-times smaller than that of the film PC-Al (2.29 nm). It can be seen that several spikes are present in the image of the PC-Al film; this can induce significant scattering losses in the nanolasers fabricated after the deposition of the insulator layer. Figure 1(c) shows the measured reflectivity spectra of the SC-Al and PC-Al films. In comparison to PC-Al, SC-Al has a higher reflectivity in the ultraviolet-to-near-infrared region. In particular, for the spectral window corresponding to wavelengths lower than 400 nm, the reflectance of SC-Al is markedly larger than that of PC-Al. This is probably owing to the ultrasmooth surface morphology of SC-Al, which lowers light absorption and prevents the random scattering caused by a rough surface and grain boundaries^39^. Figure 1(d) shows a high-resolution transmission electron microscopy (HRTEM) image of the SC-Al film after the deposition of an Al_2_O_3_ layer by the ALD method. The upper layer is the Al_2_O_3_ spacer layer and the bottom one is the SC-Al film; the interface between them is clear and sharp. The nearly perfect, atomic-like periodic array in the Al film indicates that the film is of high quality and indeed single-crystalline. The inset in Fig. 1(d) shows the electron diffraction pattern for the Al region. That the hexagonal diffraction pattern is clear and without any observable side points is indicative of the face-centered-cubic crystal structure of Al. In order to identify the crystal orientations of the Al films relative to the GaAs substrates, we perform X-ray diffraction (XRD) analyses of both Al films. Figure 2(a) shows the low-incident-angle scanning XRD pattern for the PC-Al film. As expected, diffraction peaks corresponding to Al (111), (200), and (311) surfaces are observed at 38.5°, 44.7°, and 78.2°, respectively. On the other hand, no Al-related peak is observed in the case of the SC-Al film. This is because the lattice planes of Al may not be parallel to those of the GaAs substrate. As has been reported previously^37^, because the diffraction peaks for the GaAs (100) and Al (110) surfaces are very similar, it is possible that the peak for the latter is buried in the strong signal of the former. To avoid this interference from the GaAs substrate, we examine the lattice plane Al (111), which does not exhibit radial symmetry and perform φ-dependent scanning measurements using the experimental setup shown in Fig. 2(b). Because the angle between the Al (111) and (100) planes is 34.5°, we set χ as 34.5° and 2θ as 38.5° for the φ scan. Figure 2(c) shows the results of the φ*-*dependent measurements for the PC-Al film. As expected, the peak count is not dependent on φ, owing to the random orientation of the crystals. In contrast, Fig. 2(d) shows that the count of the Al (111) peak of the SC-Al specimen is clearly dependent on φ. Note that the peak count for SC-Al is more than two orders of magnitude larger than that of PC-Al, confirming that the SC-Al film is indeed single-crystalline and of high quality.

We believe that the performance of plasmonic devices in the ultraviolet regime, such as ZnO-based MOS nanolasers, will be significantly enhanced using the superior-quality SC-Al films deposited in this study. To demonstrate that both surface roughness and metal crystallization are critical parameters for improving the performance of plasmonic nanolasers, we fabricate ZnO nanowires on the SC-Al and PC-Al films (see Fig. 3(a)); a 5-nm-thick ultrasmooth Al_2_O_3_ dielectric spacer layer is also grown by ALD (see Supporting Information). The single-crystalline ZnO nanowires are fabricated by the hydrothermal method using ZnO powder, which is dispersed directly on the patterned Al_2_O_3_/SC-Al and Al_2_O_3_/PC-Al substrates. The powder adheres to the substrates because of Van der Waals force. In contrast to the PC-Al film, the SC-Al film acts as an ultrasmooth template for growing an ultraflat Al_2_O_3_ spacer layer, which allows for adequate optical confinement and prevents the excitons generated in the ZnO nanowires from quenching rapidly on the metal surface. This allows the efficient exciton-SP energy transfer process to be sustained^7^. A scanning electron microscopy (SEM) image of a finished ZnO nanolaser is shown in Fig. 3(b). The length and hexagonal size length of the ZnO nanowire are approximately 1 μm and 30 nm, respectively. Figure 3(c) shows an HRTEM image of a ZnO nanowire taken along the 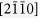 direction. The lattice fringe spacing in the HRTEM image is 0.52 nm, which corresponds to the lattice constant of the hexagonal, wurtzite-structured ZnO and indicates that the nanowires grew along the 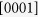 direction.

Figure 4 shows the optical characteristics of two typical nanolasers fabricated on the SC-Al and PC-Al films, as measured at 77 K. Figure 4(a) shows the emission intensity and linewidth versus pumping energy density for a 1-μm-long single ZnO nanowire on the Al_2_O_3_/SC-Al substrate. It is clearly seen that, in the log-log plot of pumping energy density and emission intensity, a nonlinear S-shaped dependence appears and, in the transition region from ~0.2 to 0.4 mJcm^−2^, a dramatic reduction in emission linewidth (from ~ 8 to ~0.2 nm) occurs. This is clear evidence of nanolaser lasing at an ultralow threshold energy density, namely, at 0.28 mJ cm^−2^. Figure 4(b) shows the corresponding pumping energy density-dependent emission spectra. For pumping energy density levels lower than the lasing threshold, a weak and broad emission peak is observed at approximately 370 nm. When the pumping energy density is increased to 0.38 mJ cm^−2^, a very narrow lasing peak appears at 371 nm. For comparison, the characteristics of the nanolaser formed on the PC-Al film and measured at 77 K and those of a 1.46-μm-long ZnO nanowire are shown in Fig. 4(c,d), respectively. In these cases too, the curves exhibit a nonlinear S-shaped section, and the linewidth narrows down to 0.3 nm; however, this occurs at a much higher threshold energy density, 10.19 mJ cm^−2^, which is 36times larger than that for the nanolaser formed on the SC-Al film. Figure 4(d) shows the measured pumping energy density-dependent emission spectra. Below the lasing threshold, a broad, spontaneous emission peak related to the ZnO nanowire is observed. For pumping energy densities higher than the threshold value, a lasing peak appears at 372 nm, in addition to the broad emission; this is the case even for a pumping energy density of 12.73 mJ cm^−2^. This suggests that the crystalline quality of the underlying metal film has a significant effect on the performance of ZnO nanolasers, as a film of high quality minimizes the metallic loss. Therefore, the SC-Al film could reduce the threshold condition and improve nanolaser performance.

To further emphasize the importance of the quality of the metal and spacer layers, we present the thresholds and yields of ZnO nanolasers fabricate on four different types of templates: Al_2_O_3_ layers formed by ALD on the PC-Al and SC-Al films and SiO_2_ layers formed by e-gun evaporation on the PC-Al and SC-Al films. All the insulating spacer layers have 5 nm thickness. The fabrication process is described in Supporting Information. The various spacer layers are characterized by AFM. The SiO_2_ spacer layer deposited by e-gun evaporation has a much rougher surface than does the Al_2_O_3_ layer grown by ALD. The optical characteristics of approximately 50 nanolasers fabricated on the four chips are measured at 77 K, in order to determine the device yield and the threshold pumping energy density. Figure 5(a) shows the numbers of the measured (blue bars) and lasing (red bars) nanolasers. Not surprisingly, the highest yield, which is 87%, is exhibited by the nanolasers formed on the SC-Al/Al_2_O_3_ template, while the lowest is exhibited by the devices fabricated on the PC-Al/SiO_2_ template. Only one out of twelve of these devices works. The second best template is SC-Al/SiO_2_, again highlighting the key role played by the metal film in determining the performance of plasmonic devices. Figure 5(b) shows the threshold pumping energy densities for all the lasing devices at 77 K. The only nanolaser fabricated on the SiO_2_/PC-Al template that works has a very high threshold (54.5 mJ/cm^2^) (black circles). The threshold pumping densities of the nanolasers fabricated on the SC-Al/SiO_2_ (red triangles) and PC-Al/Al_2_O_3_ (green diamonds) templates are similar and in the range of 10 to 30 mJ cm^−2^. The nanolasers formed on the SC-Al/ Al_2_O_3_ template have, in general, the lowest threshold pumping densities (blue stars), which range from ~0.28 to ~2 mJ cm^−2^. In the cavities of plasmonic lasers, the plasmonic loss, which can be an order of magnitude larger than the mirror loss, determines the lasing threshold^29^,^31^,^32^. As a result, the lasing threshold is highly sensitive to various morphology-related factors as well as the surface condition, variations in the metal film composition, and whether the ZnO nanowires are deformed. Even a small bump present at the interface between the ZnO nanowires and the dielectric/metal layer can increase the threshold. Therefore, in this study, the threshold did not depend significantly on the nanowire length. Comparing with the nanolasers on other templates, the threshold pumping energy density is in general one to two orders lower. This result confirms that the quality of the SC-Al/Al_2_O_3_ template prepared using MBE and ALD is very high.

Further, the ZnO lasers fabricated on this template can operate even at room temperature. Figure 6(a) shows the lasing spectra of a nanolaser fabricated on the SC-Al/ Al_2_O_3_ template and measured from 77 to 300 K. The lasing behavior of the device can be sustained till to 300 K, while its lasing wavelength red shifts from 371 to 381 nm. It is noteworthy that the threshold pumping energy density increases from 0.28 mJ cm^−2^ at 77 K to 0.84 mJ cm^−2^ at 273 K; this corresponds to a characteristic temperature (T_0_) of approximately 178 K. A T_0_value this large is mainly attributable to the high-quality template, which lowers the metallic losses. The Fermi temperature of metals is high. Thus, the nanolaser threshold increases with the temperature, owing to the electron-phonon scattering losses in the metal layer and the ZnO nanowire. Since the optical gain of plasmonic nanolasers depends on the generation of excitons in the ZnO nanowire, the dependence of the position of the lasing peak would follow the Varshni trend exhibited by excitons in ZnO. The dilation of the lattice and the electron-lattice interactions that occur with an increase in the temperature cause the observed redshift and decrease the dipole oscillator strength in exciton forms. The exciton binding energy of ZnO is approximately 60 meV; thus, the emission is the result of the excitonic gain from 77 to 300 K. Figure 6(b) shows the emission intensity and linewidth versus the pumping energy density curves for a 1-μm-long ZnO nanowire formed on the SC-Al/Al_2_O_3_ template, as determined at 300 K. Clear lasing features, such as an S-shaped response curve, a threshold pumping energy density of 6.1 mJ cm^−2^, and a dramatic decrease in the linewidth to 0.3 nm, are observed. The inset of Fig. 6(b) shows that the emission is highly polarized along the direction parallel to the nanowire axis, with the degree of polarization being 75%; this indicates that the longitudinal lasing mode is still the fundamental SP mode.

In conclusion, we successfully synthesize a high-performance plasmonic nanolaser that operates in the ultraviolet regime. The interfacial roughness and, in particular, the quality of the metal film play a key role in determining the performance of the ZnO nanolasers. We use MBE to grow high-quality single-crystalline Al films and subsequently form an ultrasmooth Al_2_O_3_ layer by ALD. The ZnO nanowire is formed by the hydrothermal method. Thus, we could realize an ultraviolet plasmonic nanolaser with a very low threshold pumping energy density and a high characteristic temperature. The nanolaser can operate at room temperature and exhibits distinct lasing characteristics. This study highlights the importance of the quality of the metal film as well as interface control with respect to plasmonic device performance and should pave the way for the use of ultraviolet nanolasers in various applications.

## Methods

### Device fabrication

The ultraviolet plasmonic nanolasers are fabricated by forming ZnO nanowires on 100-nm-thick Al films with a 5 nm-thick insulating spacer layer in a MOS structure. For comparison, a single-crystalline Al (SC-Al) grown by MBE and a polycrystalline Al (PC-Al) film deposited by e-gun evaporation are used as the initial templates. The details of the fabrication processes for the Al films are given in Supporting Information. The Al_2_O_3_ and SiO_2_ insulating spacer layers are grown by ALD and e-gun evaporation, respectively.

### Measurements

To confirm that the measured optical signals correspond to a single ZnO nanowire and that there is no interference from any of the nanowires nearby, we first use SEM to determine the locations of patterned squares with a single ZnO nanowire. The patterned substrates are placed in a cryogenic vacuum chamber, which could be cooled to 77 K using liquid nitrogen. The targeted patterned squares with a single ZnO nanowire are then re-identified using a charge-coupled device (CCD) camera. Next, the ZnO nanowire is optically pumped by the third harmonic generation of a Nd: YVO_4_ 355 nm pulse laser with a pulse duration of 0.5 ns and repetition rate of 1 kHz. By using a 100× near-ultraviolet infinity-corrected objective lens with a numerical aperture of 0.55 (Mitutoyo), the pumping beam is made normally incident onto the patterned square; the diameter of the pumping beam is 15 μm. The reflected light is collected using a 600 μm core ultraviolet optical fiber and detected with a liquid-nitrogen-cooled CCD camera attached to a 320 mm single monochromator (HR320, JobinYvon) with a spectral resolution of approximately 0.2 nm. Two emission spots are observed below the threshold density using the CCD camera; the connection between the two emission points represents the direction of the ZnO nanowire. A polarizer is placed in front of the fiber to determine the degree of polarization. The degree of polarization is defined by (*I*_*max*_−*I*_*min*_)/(*I*_*max*_ + *I*_*min*_), where *I*_*max*_ and *I*_*min*_ are the maximum and minimum intensities of the lasing peak. For time-resolved photoluminescence (PL) experiments, a 266 nm laser pulse generated by tripling the frequency of a Ti: sapphire laser (Mira 900, Coherent) with a repetition rate of 76 MHz and pulse duration of 200 fs is used as the probe beam. The PL signal emitted from the sample is collected by a 50× plan ultraviolet infinity-corrected objective lens and fed into a streak camera (Hamamtsu,C5680) with a temporal resolution of approximately 6 ps in the single-shot mode.

### Simulation

Two-dimensional device simulations are performed using the COMSOL RF module. We use the eigenvalue solver for the finite-element-method to determine the eigenmodes of the ZnO nanowire formed on the dielectric/metal substrate. The computation domains are enclosed by perfectly matched layers, in order to absorb the scattered power with minimum reflection. For the simulations, the refractive indices of ZnO, Al_2_O_3_, and SiO_2_ are taken to be 2.54, 1.79, and 1.47, respectively. The simulation details and the refractive index of aluminum, as determined from the measured reflectivity spectra, are listed in Supporting Information.

## Additional Information

**How to cite this article**: Chou, B.-T. *et al*. Single-crystalline aluminum film for ultraviolet plasmonic nanolasers. *Sci. Rep.* **6**, 19887; doi: 10.1038/srep19887 (2016).

## Supplementary Material

Supplementary Information

## Figures and Tables

**Figure 1:**
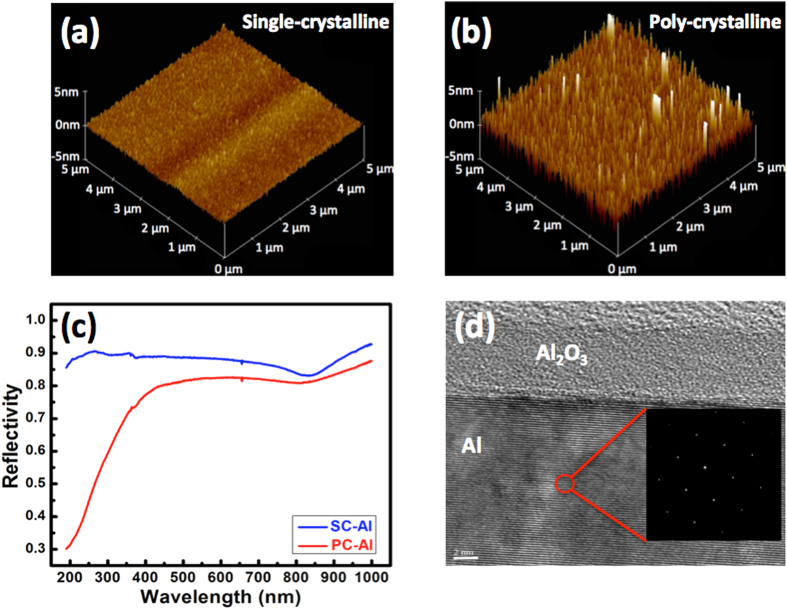
(**a**,**b**) 5 × 5 μm^2^ top-view atomic force microscope images of the single- and polycrystalline Al films, respectively. (**c**) Reflectivity spectra of the twoAl films. (**d**) Cross-sectional transmission electron microscopy image of the Al_2_O_3_ layer on the single-crystalline Al film. Insetin (**d**) electron diffraction pattern of the Al region.

**Figure 2:**
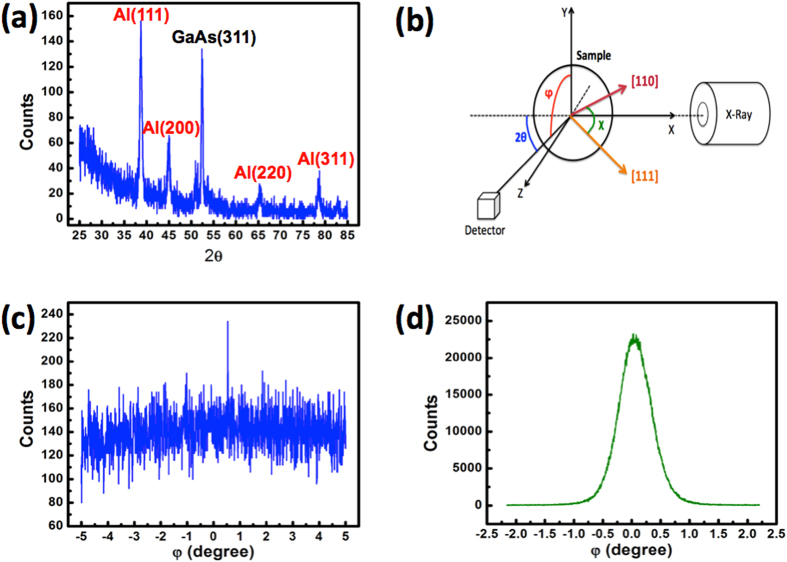
(**a**) Low-incident-angle 2θ scan of the polycrystalline Al film. (**b**) XRD setup for the φ*-*dependent measurement of the Al(111) plane (**c**) Results of the φ*-*dependent scan of the polycrystalline Al film. (**d**) Results of the φ*-*dependent scan of the single-crystalline Al film.

**Figure 3:**
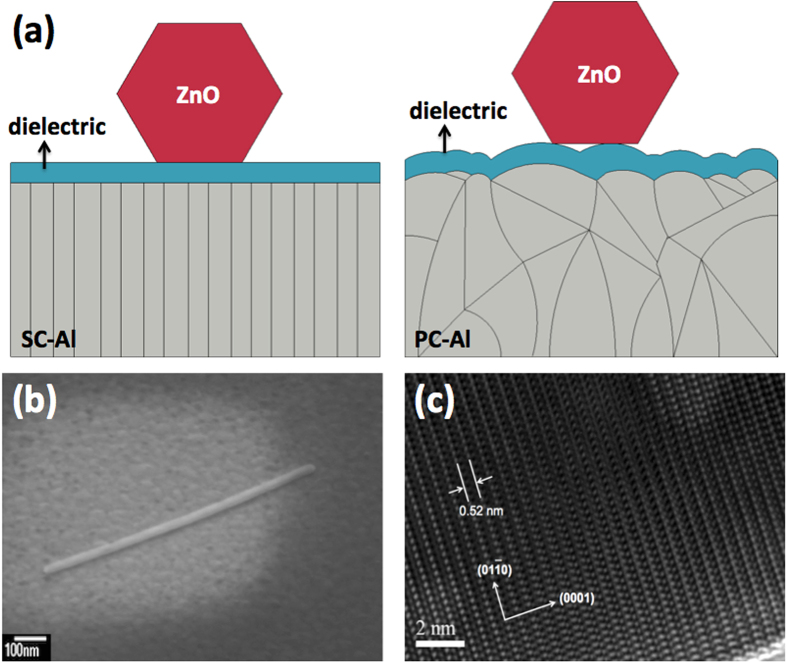
(**a**) Schematics of the ZnO nanowires formed on the tops of the single- and polycrystalline Al films with a dielectric spacer layer. (**b**) Scanning electron microscopy image of a ZnO plasmonic nanolaser. The length and hexagonal side length of the ZnO nanowire are 1 μm and 30 nm, respectively. (**c**) High-resolution transmission electron microscopy image of a ZnO nanowire taken along the 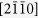 direction.

**Figure 4:**
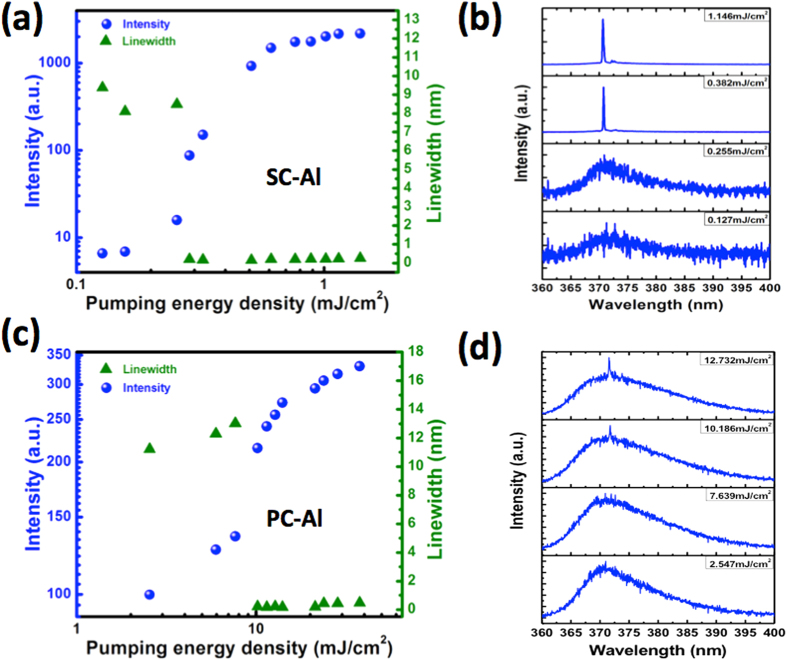
Emission intensity and linewidth as functions of the pumping energy density (**a**) for a nanolaser formed on the SC-Al/Al_2_O_3_template and (**b**) the corresponding emission spectrum. Emission intensity and linewidth as functions ofthe pumping energy density (**c**) for a nanolaser formed on the PC-Al/Al_2_O_3_template and (**d**) the corresponding emission spectrum. All the data are measured at 77 K.

**Figure 5:**
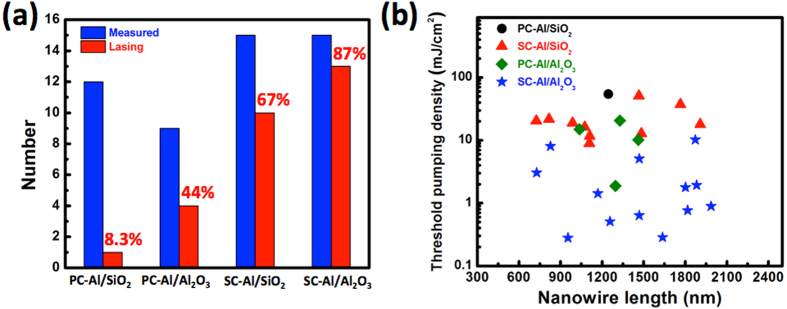
(**a**) Numbers of the measured and lasing nanowires corresponding to the four kinds of templates.(**b**) Threshold pumping energy density as a function of nanowire length for the four kinds of specimens. All the data are measured at 77 K.

**Figure 6:**
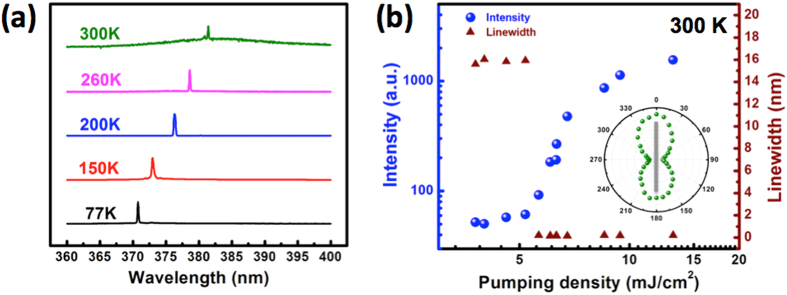
(**a**)Temperature-dependent lasing spectra of a nanolaser formed on the SC-Al/Al_2_O_3_template, as measured from 77 to 300 K. (**b**) Emission intensity and line width versus pumping energy density of the nanolaser at 300 K. Inset in (**b**) corresponding lasing polarization plot.
