# Supplementary material for: Single-crystalline aluminum film for ultraviolet plasmonic nanolasers
Source: Sci Rep. 2016 Jan 27;6:19887. doi: 10.1038/srep19887 (PMC4728607; doi:10.1038/srep19887)
Supplement: Supplementary Information [file srep19887-s1.pdf]

Supporting Information

Single-crystalline aluminum film for ultraviolet plasmonic nanolasers

Bo-Tsun Chou[1], Yu-Hsun Chou[2, 3], Yen-Mo Wu[3], Yi-Cheng Chung[4], Wei-Jen Hsueh[5], Shih-Wei Lin[1], Tien-Chang Lu[3], Tzy-Rong Lin[4, 6] and Sheng-Di Lin [1*]

1. *Department of Electronics Engineering, National Chiao Tung University, Hsinchu, Taiwan*
2. *Institute of Lighting and Energy Photonics, National Chiao Tung University, Tainan, Taiwan*
3. *Department of Photonics, National Chiao Tung University, Hsinchu, Taiwan*
4. *Department of Mechanical and Mechatronic Engineering, National Taiwan Ocean University, Keelung, Taiwan*
5. *Department of Electrical Engineering, National Central University, Chungli, Taiwan*
6. *Institute of Optoelectronic Sciences, National Taiwan Ocean University, Keelung*

*E-mail address: sdlin@mail.nctu.edu.tw

Outline

1. **Nanolaser fabrication** **3**
2. **Complex dielectric constants extraction for the two Al films** **4**
3. **Nanolaser numerical simulation** **7**
4. **Experimental results of nanolasers with SiO_2 spacer layer** **9**
5. **References** **11**

Supplementary Discussions

1. Nanolaser fabrication

A. Single-crystalline Al films epitaxy:

The single-crystalline Al films were grown on a GaAs (100) substrate by Varian Gen II solid-source molecular beam epitaxy system (MBE). We first grew a 200-nm-thick undoped GaAs buffer layer serve as the template for the following Al growth. The surface was turned from As-rich into Ga-rich before cooling down to room temperature. The wafer was then kept in the ultra-high vacuum chamber to prevent the surface from oxidation. When the residual arsenic pressure was pumped down to less than $1x10^{-10}$ torr, a 100-nm-thick Al layer was grown at ~ 0 °C with a growth rate of 0.05 nm/s.

B. Poly-crystalline Al films evaporation:

The poly-crystalline Al films were evaporated on the GaAs (100) substrate by an e-gun evaporator. Before the GaAs substrate was loaded into the evaporator chamber, a de-oxidation process using HCl etching solution was performed to remove the native oxide on the GaAs surface. After the standard clean process, the GaAs substrate had been loading in to the electron-gun chamber immediately. As the chamber pressure was less than 3×10^{-6} torr, we evaporated a 100-nm-thick Al film at room temperature with an evaporation rate of 0.3 nm/s.

C. Al_2O_3 dielectric spacer layer growth:

The dielectric 5-nm-thick Al_2O_3 spacer layers were grown on Al films by atomic layer deposition (ALD) system (Cambridge NanoTech Fiji-202 DCS). The deposition used TMA ($Al(CH_3)_3$), H_2O and O_2 as precursors and N_2 as the carrier/purge gas and performed at 250 °C. Each reaction cycle consisted of four steps: oxygen precursor injection, N_2 purge, TMA injection, and N_2 purge. After repeated 54 reaction cycles, a 5-nm-thick Al_2O_3 dielectric spacer layer was deposited on Al/GaAs substrates.

D. SiO_2 dielectric spacer layer evaporation:

The 5-nm-thick SiO_2 dielectric spacer layers were deposited by e-gun evaporation system (ULVAC EBX-8C) on Al films. The purity of the SiO_2 solid source was 99.99% provided by Gredmann Corporation. Before the evaporation, the chamber pressure was about 3×10^{-6} Torr and the growth rate was about 0.02 nm/s at 20 °C. The evaporation rate and the film thickness were monitored in real time with a quartz crystal oscillator.

2. Complex dielectric constants extraction for the two Al films

The optical properties such as absorption, refraction and transmission of material can be described by its complex dielectric constants. As shown in Figure 3 (c) in the main text, the reflectivity of the PC-Al film is lower than that of the SC-Al one, especially in the wavelength range below 450 nm. To quantitatively explain the reflectivity discrepancy, we extracted the optical parameters of these two films by

fitting the measured reflectivity spectra with the Drude-Lorentz model. The complex dielectric constant for metals can be expressed as below [1, 2, 3]

$$\varepsilon_{DL}(\omega) = \varepsilon_{\infty} - \frac{\omega_p^2}{\omega^2 + i\omega\gamma_p} + \sum_{i=1}^{2} \frac{\Delta\varepsilon_i \omega_{L,i}^2}{\omega^2 - \omega_{L,i}^2 - i\omega\gamma_{L,i}} \tag{1}$$

where ω_p and ω_L are the plasmon resonance frequency of Al and Lorentz model resonance frequency, respectively. ω is the angular frequency of the incident light. ε_{∞} is the background permittivity. γ_p and γ_L are the damping coefficients in Drude and Lorentz models, respectively. The refractive index n and κ can then be obtained accordingly by the following relations.

$$n^2 = \frac{1}{2}\left[\mathrm{Re}\left(\varepsilon_{DL}(\omega)\right) + \sqrt{\left[\mathrm{Re}\left(\varepsilon_{DL}(\omega)\right)\right]^2 + \left[\mathrm{Im}\left(\varepsilon_{DL}(\omega)\right)\right]^2}\right] \tag{2}$$

$$\kappa^2 = \frac{1}{2}\left[-\mathrm{Re}\left(\varepsilon_{DL}(\omega)\right) + \sqrt{\left[\mathrm{Re}\left(\varepsilon_{DL}(\omega)\right)\right]^2 + \left[\mathrm{Im}\left(\varepsilon_{DL}(\omega)\right)\right]^2}\right] \tag{3}$$

And the reflectivity (R) can be calculated from Eqs. (2) and (3) with

$$R = \frac{(n-1)^2 + \kappa^2}{(n+1)^2 + \kappa^2} \quad . \tag{4}$$

By using Eq. (4), we fitted the whole spectrum range of Figure 3 (c) in the main text. The fitting parameters and their values are listed in Table S1.

Table S1. Drude-Lorentz fitting parameters of SC-Al and PC-Al films.

ω and γ unit: rad/s

Sample	ε_∞	ω_p	γ_p	$\Delta\varepsilon_1$ / $\omega_{L,1}$ / $\gamma_{L,1}$	$\Delta\varepsilon_2$ / $\omega_{L,2}$ / $\gamma_{L,2}$
SC-Al	2.1	2.4×10^{16}	1.0×10^{15}	4.0	3.5
				2.4×10^{15}	2.5×10^{15}
				3.5×10^{14}	8.0×10^{14}
PC-Al	5.9	2.4×10^{16}	1.9×10^{15}	6.0	1.2
				2.0×10^{15}	2.3×10^{15}
				4.0×10^{14}	3.8×10^{14}

The fitting results are plotted in Figure S1. Figure S1 (a) shows the SC-Al fitting result. The measured reflectivity spectrum (red) can be well fitted by Drude-Lorentz model fitting equation (blue) in the whole spectrum range. However, as Figure S1 (b) shows, clear discrepancy is spotted between model fitting and measured reflectivity spectra of PC-Al film for less than 400 nm, probably because of the grain boundaries and the surface roughness of PC-Al sample.

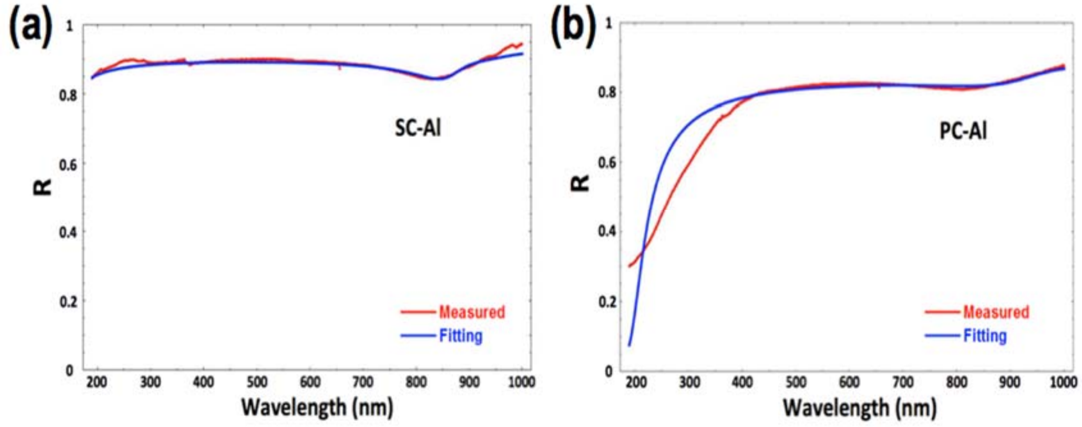

Figure S1. (a) Measured and fitting reflectivity spectra for SC-Al film. (b) Measured and fitting reflectivity spectra for PC-Al film.

With the obtained fitting parameters of SC-Al, we calculated the complex dielectric constants of the SC-Al films by using Eqs. (1)-(3). The real and imaginary parts of the obtained dielectric constants are plotted in Figures S2 (a) and (b), respectively. For comparison, the dielectric constants measured with bulk Al sample by Rakic *et. al.* [S1] are also plotted. Figure S2 (a) shows the real part of complex dielectric constant of our SC-Al film is very close to theirs but, as shown in Figure S2 (b), our imaginary part is slightly higher.

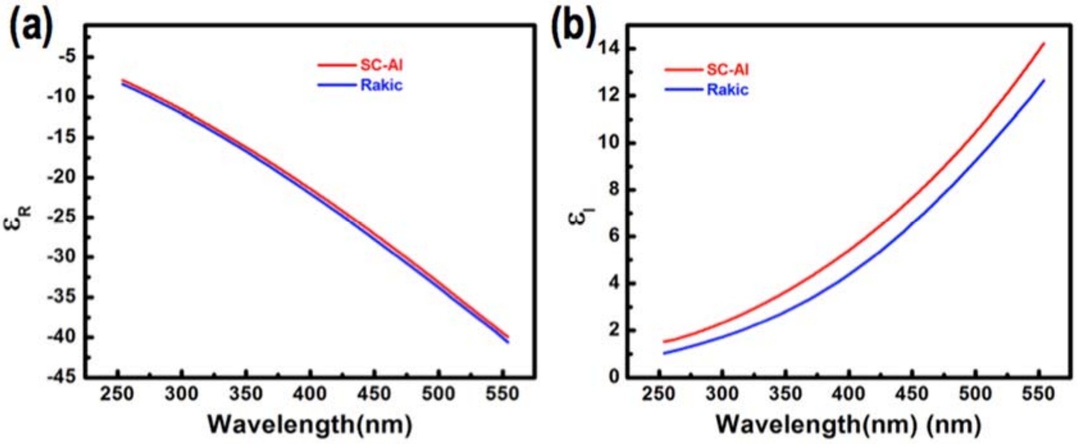

Figure S2 (a) Real part of complex dielectric constant of our SC-Al film and of those in Ref. [S1] as a function of wavelength. (b) Imaginary part of complex dielectric constant of our SC-Al film and of those in Ref. [S1] as a function of wavelength.

3. Nanolaser numerical simulation

We used the commercial software COMSOL, the mode solver of the finite-element method (FEM) package, to find the eigenmodes of the ZnO plasmonic

nanolasers. The surface plasmon propagation length L_p, describing how long the surface plasmon mode can propagate, is determined with the imaginary part of the modal propagation constant k_z as $L_p = \left[2\,\mathrm{Im}\{k_z\}\right]^{-1}$ [4, 5, 6, 7]. The waveguide confinement factor Γ_{wg} is defined as the ratio of the modal gain to the material gain in the active region, expressed as $\Gamma_{wg} = \frac{\frac{n_a}{2\eta_0}\int_{A_a} d\rho \left|\mathbf{E}(\rho)\right|^2}{P_z}$, where P_z is the power flow in the propagation direction; n_a is the refractive index of the gain medium; A_a is the cross-section area of the gain medium; and η_0 is the intrinsic impedance. [4]. In MOS nanolasers, the thickness of spacer oxide layer is a critical parameter. Figure S3 (a) shows the simulation results of surface plasmon propagation length and waveguide confinement factor as a function of Al_2O_3 thickness from 3 to 20 nm for ZnO nanowire lying on the SC-Al film. A thinner spacer thickness h_g leads to a stronger coupling between the guided modes of the nanowire and the surface plasmon modes of the planar structure, resulting in the good confinement of the fundamental mode. However, for h_g below 7 nm, the field distribution of the hybrid modes is too close to the metallic and gain region so the metal intrinsic ohmic damping loss increases and the propagation length shortens. Therefore, considering the native oxide of aluminum film, we used 5-nm-thick Al_2O_3 spacer layer for our nanolasers.

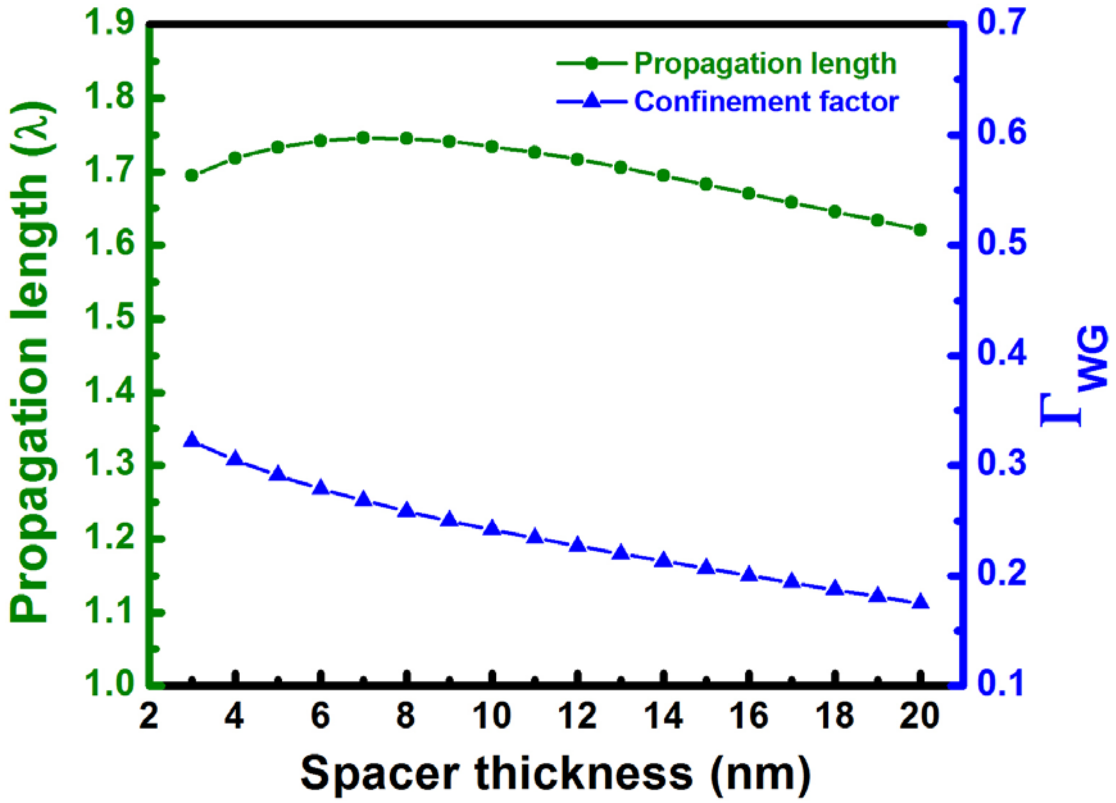

Figure S3 (a). Calculate surface plasmon propagation length, and waveguide confinement factor of surface plasmon modes as a function of spacer thickness h_g. The side length is d = 30 nm for the ZnO nanowire on the Al_2O_3/SC-Al substrate.

As the Figure S3 (b) shows, once the one-side length (d) of the hexagonal cross-section of the ZnO nanowire is below 55 nm, only the fundamental plasmonic mode can sustain in the cavity. Since the typical one-side hexagon length of our ZnO nanowire was 30 nm, only the F mode can be observed in our case. In addition, far-field polarization measurement in the Fig. 6 (b) of main text showed that the polarization direction was along the nanowire, which is the distinct feature of the fundamental surface plamonic mode in our nanolaser structures.

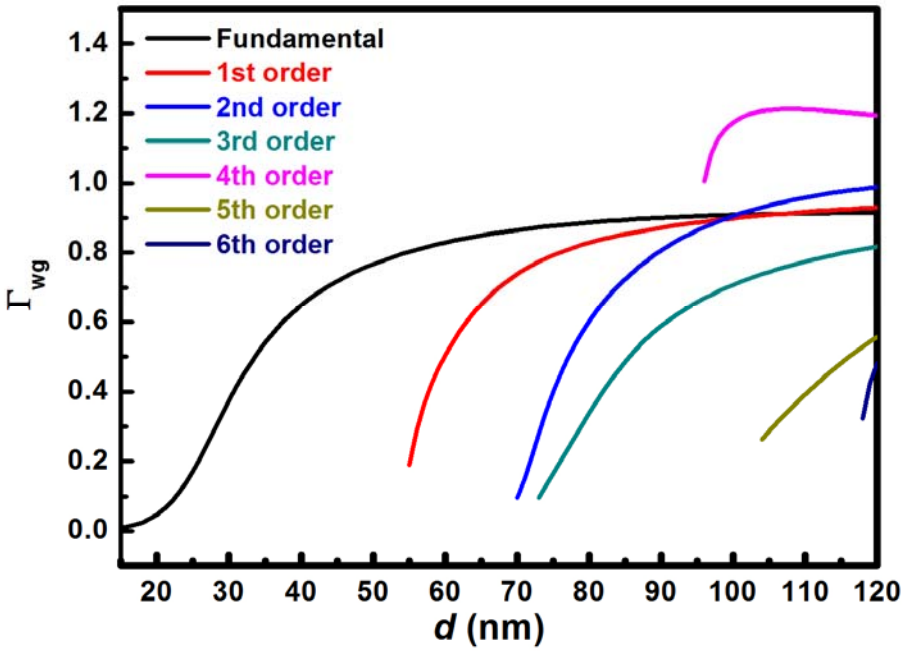

Figure S3 (b). The confinement factors of various plasmonic cavity modes.

4. Experimental results of nanolasers with SiO_2 spacer layer

Figure S4 (a) shows the AFM surface morphology of a nanolaser template consisting of PC-Al film and a 5-nm-thick SiO_2 spacer layer. The surface is quite rough with an RMS roughness of 2.14 nm. Figure S4 (b) shows the measured power-dependent emission spectra from a nanolaser at 77 K, indicating a lasing threshold power density of 54.5 mJ cm^{-2}. The inset shows that, below the lasing threshold, a broad emission spectrum of the 1.24-μm-long ZnO nanowire was observed. A clear lasing peak at 368 nm with the linewidth narrowing down to 0.8 nm was seen above threshold. Owing to the rough surface morphology, the SPs suffer serious extrinsic scattering loss and intrinsic ohmic damping loss. Even more importantly, the rough surface could

drastically reduce the coupling efficiency between the excitons and SPs so a very high threshold power density was obtained.

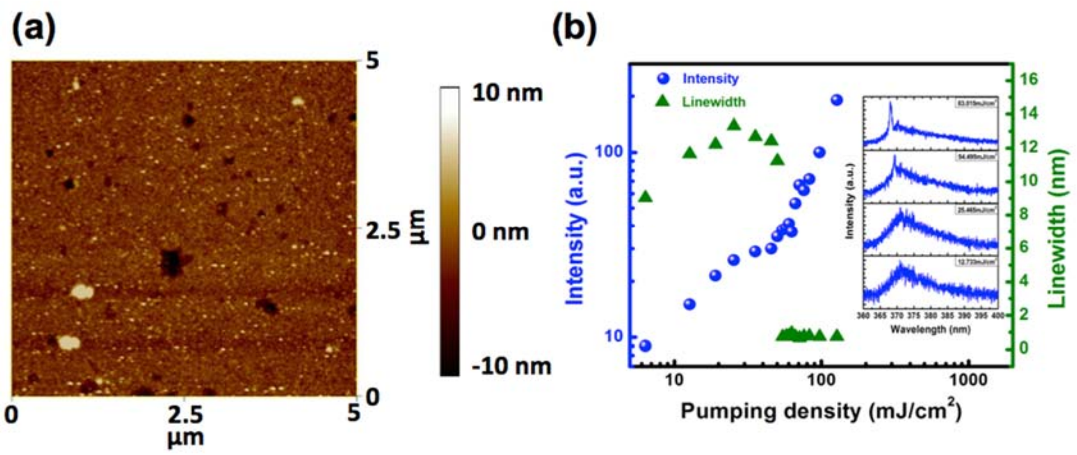

Figure S4. (a) AFM surface morphology of 5-nm-thick SiO_2 deposited on PC-Al film. (b) Emission intensity and linewidth versus pumping density of the SiO_2/PC-Al nanolasers at 77 K. Inset: 77K power-dependent spectrum under various power densities.

Figure S5 (a) shows the surface morphology of a nanolaser template consisting of SC-Al film and a 5-nm-thick SiO_2 spacer layer. The RMS roughness is 1.64 nm, slightly better than the previous one. Figure S5 (b) shows the measured power-dependent emission spectra from a 1.11-μm-long ZnO nanolaser at 77 K. The SC-Al film could reduce the SPs intrinsic damping loss and grain boundaries scattering loss but the surface roughness caused considerable extrinsic SPs scattering loss. The inset to the right of Figure S3 shows the fluctuated spontaneous and lasing spectra with two obvious lasing peaks at 369 and 371 nm. The integrated emission intensity suggests a lasing threshold power density of 10.5 mJ cm^{-2}, which is about 5-fold smaller than ZnO nanowire lying on the PC-Al/SiO_2 template.

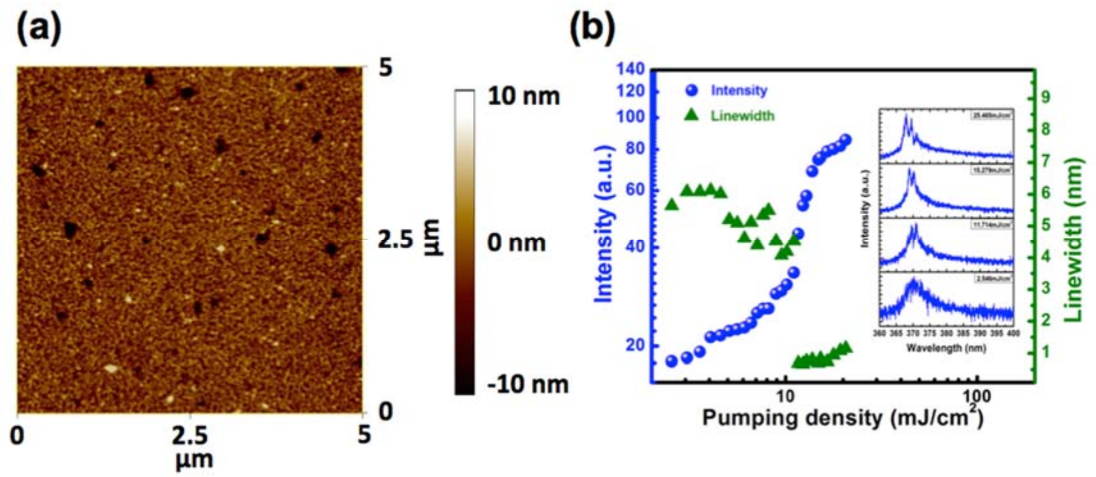

Figure S5. (a) AFM surface morphology of 5-nm-thick SiO_2 deposited on SC-Al film. (b) Emission intensity and linewidth versus pumping density of the SiO_2/SC-Al nanolasers at 77 K. Inset: 77K power-dependent spectrum under various power densities.

References

1. Chen W, Thoreson MD, Ishii S, Kildishev AV, Shalaev VM. Ultra-thin ultra-smooth and low-loss silver films on a germanium wetting layer. *Optics Express* **18**, 5124-5134 (2010).

2. Hyuk Park J, Nagpal P, Oh S-H, Norris DJ. Improved dielectric functions in metallic films obtained via template stripping. *Appl Phys Lett* **100**, 081105 (2012).

3. Rakić AD, Djurišić AB, Elazar JM, Majewski ML. Optical properties of metallic films for vertical-cavity optoelectronic devices. *Appl Opt* **37**, 5271-5283 (1998).

4. Chou Y-H*, et al.* Ultrastrong Mode Confinement in ZnO Surface Plasmon Nanolasers. *ACS Nano* **9**, 3978-3983 (2015).

5. Zhang Q*, et al.* A room temperature low-threshold ultraviolet plasmonic nanolaser. *Nature communications* **5**, (2014).

6. Oulton RF*, et al.* Plasmon lasers at deep subwavelength scale. *Nature* **461**, 629-632 (2009).

7. Sidiropoulos TP, *et al.* Ultrafast plasmonic nanowire lasers near the surface plasmon frequency. *Nature Physics* **10**, 870-876 (2014).
